# Supplementary material for: Evaluating the Nutritional Properties of Food: A Scoping Review
Source: Nutrients. 2022 Jun 5;14(11):2352. doi: 10.3390/nu14112352 (PMC9182956; doi:10.3390/nu14112352)
Supplement: Supplementary file 1 [file nutrients-14-02352-s001.zip › Table S1 The detailed search strategies and results in the databases-The detailed search strategies and results in the databases.pdf]

Table S1 The detailed search strategies and results in the databases

Database	Search strategies	Results
Web of science	(((TS=(Food Quality)) OR TS=(Food Qualities)) OR TS=(Qualities, Food)) OR TS=(Quality, Food) AND TS=(Diet, Food, and Nutrition) AND (((TS=(Nutrients)) OR TS=(Nutrient)) OR TS=(Macronutrients)) OR TS=(Macronutrient) AND (((TS=(evaluation)) OR TS=(evaluate tool)) OR TS=(evaluation method)) OR TS=(evaluation indicator) AND TS=(method)	3004 items
PubMed	(((((food[MeSH Terms]) AND (Food Quality[MeSH Terms])) AND (Diet, Food, and Nutrition[MeSH Terms])) AND ((((evaluation) OR (evaluation method)) OR (evaluation tool)) OR (evaluation indicator))) AND (nutrients[MeSH Terms])) AND (method)	201 items
Scopus	((TITLE-ABS-KEY (food AND quality) OR TITLE-ABS-KEY (food AND qualities) OR TITLE-ABS-KEY (qualities, AND food) OR TITLE-ABS-KEY (quality, AND food))) AND (TITLE-ABS-KEY (diet, AND food, AND nutrition)) AND ((TITLE-ABS-KEY (nutrients) OR TITLE-ABS-KEY (nutrient) OR TITLE-ABS-KEY (macronutrients) OR TITLE-ABS-KEY (macronutrient))) AND ((TITLE-ABS-KEY (evaluation) OR TITLE-ABS-KEY (evaluation AND tool) OR TITLE-ABS-KEY (evaluation AND method) OR TITLE-ABS-KEY (evaluation AND indicator))) AND (TITLE-ABS-KEY (method))	169 items
